# Evaluation of an Internet-Delivered Multidisciplinary Pain Acceptance and Commitment Therapy (IMPACT) Program for Individuals Living with Chronic Pain: A Feasibility and Pilot Study

**DOI:** 10.1080/24740527.2026.2696248

**Published:** 2026-08-18

**Authors:** Renée El-Gabalawy, Brigitte Sabourin, Gregg A. Tkachuk, Gabrielle S. Logan, Kailey E. Penner, Barbara Shay, Elena Bilevicius, Lesley A. Graff, Ryan Skrabek, Jessica Senn, Steve Yurkiw, Ryan Amadeo, Jori Ganetsky, John Walker

**Affiliations:** a Max Rady College of Medicine, University of Manitoba, Winnipeg, Manitoba, Canada; b Department of Clinical Health Psychology, University of Manitoba, Winnipeg, Manitoba, Canada; c Department of Anesthesiology, Perioperative and Pain Medicine, University of Manitoba, Winnipeg, Manitoba, Canada; d Department of Psychology, University of Manitoba, Winnipeg, Manitoba, Canada; e Department of Psychiatry, University of Manitoba, Winnipeg, Manitoba, Canada; f CancerCare Manitoba, Winnipeg, Manitoba, Canada; g Cognitive Behaviour Institute of Manitoba, Winnipeg, Manitoba, Canada; h Department of Physiotherapy, School of Medical Rehabilitation, University of Manitoba, Winnipeg, Manitoba, Canada; i Inflammatory Bowel Disease Clinical and Research Centre, University of Manitoba, Winnipeg, Manitoba, Canada; j Section of Rehabilitation Medicine, Department of Internal Medicine, University of Manitoba, Winnipeg, Manitoba, Canada; k Office of Innovation and Scholarship in Medical Education, University of Manitoba, Winnipeg, Manitoba, Canada; l Department of Community Health Sciences, University of Manitoba, Winnipeg, Manitoba, Canada

**Keywords:** ACT, Acceptance and Commitment Therapy, chronic pain, self-directed, internet-based intervention, digital health, eHealth

## Abstract

**Background:**

Chronic pain affects approximately 20% of Canadians and has profound health impacts, highlighting the need for accessible, evidence-based interventions like Acceptance and Commitment Therapy (ACT). Internet-based, self-directed programs may enhance access, though few have been tested in Canadian chronic pain populations.

**Aims:**

The primary objective involved assessing the feasibility of the IMPACT (Internet-delivered Multidisciplinary Pain Acceptance and Commitment Therapy) program, a 10-module, self-directed intervention co-developed by patient-partners and pain experts. Secondary objectives included examining pilot outcomes (pain and mental health).

**Methods:**

Adults referred for outpatient chronic pain care were invited to participate. Feasibility was assessed via participant ratings on Likert scales and open-ended feedback. Pain-related (e.g., catastrophizing, interference) and mental health-related (e.g., anxiety and depression) outcomes were assessed using validated measures at baseline, post-intervention, and at 6-month follow-up.

**Results:**

Of the 115 who expressed interest (25.2% response rate), 76 consented, and 68 activated the online program (89.5% participation rate). On average, 6.44 program modules (*SD *= 3.82) were completed out of 10 (7 core, 3 optional). Most rated the program as user-friendly (80.0%) and clear (92.0%). Time commitment was the most common barrier to participation. Pilot analyses suggested significant improvements across domains at the 6-month follow-up. Large effect sizes were observed for improvements in pain interference (*g* = −0.92), catastrophizing (*g* = −0.95), and psychosocial impact (*g* = −1.06) among full or partial program completers.

**Conclusions:**

An internet-based, self-directed ACT program appears feasible, with encouraging pain and psychosocial impact trends. The IMPACT program is currently widely available for free to Canadians through the Power Over Pain portal.

## RÉSUMÉ

**Contexte :** La douleur chronique touche environ 20 % de la population canadienne et entraîne d’importantes répercussions sur la santé, ce qui met en lumière la nécessité d’offrir des interventions accessibles et fondées sur des données probantes, comme la thérapie d’acceptation et d’engagement (ACT). Les programmes autoguidés offerts en ligne pourraient améliorer l’accès à ces interventions, mais peu d’entre eux ont été évalués auprès de personnes vivant avec de la douleur chronique au Canada.

**Objectifs :** L’objectif principal était d’évaluer la faisabilité du programme IMPACT (Internet-delivered Multidisciplinary Pain Acceptance and Commitment Therapy), une intervention autoguidée en 10 modules conçue conjointement par des patients partenaires et des spécialistes de la douleur. Les objectifs secondaires visaient à examiner les résultats préliminaires du projet pilote en matière de douleur et de santé mentale.

**Méthodes :** Des adultes orientés vers des soins ambulatoires pour la douleur chronique ont été invités à participer. La faisabilité a été évaluée à partir des réponses des participants à des échelles de Likert et de leurs commentaires libres. Les résultats liés à la douleur, notamment le catastrophisme et l’interférence de la douleur, ainsi que ceux liés à la santé mentale, notamment l’anxiété et la dépression, ont été évalués au moyen d’outils de mesure validés au début de l’étude, après l’intervention et lors du suivi à six mois.

**Résultats :** Parmi les 115 personnes ayant manifesté leur intérêt (taux de réponse de 25.2 %), 76 ont consenti à participer et 68 ont activé le programme en ligne (taux de participation de 89.5 %). En moyenne, les participants ont terminé 6.44 des 10 modules du programme (ÉT = 3.82), soit sept modules de base et trois modules facultatifs. La plupart ont jugé le programme convivial (80.0 %) et clair (92.0 %). Le temps à consacrer au programme constituait l’obstacle à la participation le plus souvent signalé. Les analyses préliminaires ont révélé des améliorations significatives dans tous les domaines évalués lors du suivi à six mois. Chez les participants ayant suivi le programme en totalité ou en partie, des effets de grande ampleur ont été observés sur l’interférence de la douleur (g = –0.92), le catastrophisme (g = –0.95) et les répercussions psychosociales (g = –1.06).

**Conclusions :** Un programme autoguidé de thérapie d’acceptation et d’engagement offert en ligne semble réalisable et présente des tendances encourageantes quant à la douleur et aux répercussions psychosociales. Le programme IMPACT est actuellement largement accessible, sans frais, aux personnes vivant au Canada par l’intermédiaire du portail Power Over Pain

## Introduction

Chronic pain is a complex health condition with well-documented negative sequalae and impairments across several psychosocial domains.^1,2^ Chronic pain affects approximately 20% (over 7.6 million) of Canadians at some point in their lives throughout their lives,^3^ and an estimated one billion people worldwide.^4^ These numbers are expected to rise substantially with population growth and aging.^3^ As one of the leading causes of disability,^5^ chronic pain is associated with significant personal and societal costs – estimated at $40 billion annually in direct and indirect expenses in Canada alone.^3^ The multifactorial nature of chronic pain often leads to significant biopsychosocial impacts, including interference with daily activities,^2,6–8^ increased mental health concerns,^1,9,10^ heightened risk of suicide,^11^ and insomnia,^12^ underscoring the urgent need for effective treatment options.

Despite the well-documented detrimental effects of chronic pain, treatment availability in Canada is limited.^13^ It is not uncommon for individuals with chronic pain to wait more than 5 years for access to specialized chronic pain services.^13^ Two systematic reviews demonstrated that waiting for pain treatment can lead to either static or worsening physical health and mental health, with some studies reporting negative impacts as early as 5 weeks post-referral.^14,15^ This prompted the development of a national benchmark recommendation by the Canadian Pain Society of providing clinical services within 6 months of referral.^16^ Locally, data from a sample of 100 new pain clinic patients in Manitoba, Canada revealed that 43.0% waited over a year for an appointment, and 34.0% waited over 2 years.^7^ Similar wait times were found across Ontario and Québec.^7,^ These delays in accessing services highlight the need to expand the provision of timely and accessible evidenced-based care for individuals living with chronic pain.

### Acceptance and Commitment Therapy

With the rise of chronic pain and known negative consequences, there has been increasing focus on understanding best management practices. According to the Canadian Pain Task Force, multidisciplinary care, which includes psychological treatment, is the most effective approach to chronic pain management.^17,^ Cognitive-Behavioral Therapy (CBT) has been well-validated as an effective approach among those with chronic pain,^18,19^ and commonly takes a more symptom-focused, change-oriented approach. Acceptance and Commitment Therapy (ACT), a third-wave CBT, has increasingly been examined within chronic pain samples, and has demonstrated significant benefit.^20^ ACT for chronic pain is characterized by a more general, non-symptom focused approach to improve quality of life (QOL). In ACT, there is less emphasis on attempts at controlling or eliminating pain; it instead shifts the focus to increasing engagement in value-consistent behaviors even in the presence of pain, with the goal of improving psychological flexibility and overall well-being.^21^ ACT interventions typically teach skills across six core domains: willingness to experience difficult internal states, contact with the present moment, perspective-taking, value clarification, cognitive defusion (distancing from unhelpful thoughts), and committed action (in alignment with personal values).^22^ Systematic reviews and meta-analyses support the significant benefits and effectiveness of ACT for chronic pain across multiple outcomes,^21,23–25^ including depression, QOL, and pain interference, persisting several months post-intervention.^26^

### Internet-based interventions for chronic pain

Internet-based pain management resources have proliferated in recent years. They have become an important option for individuals with chronic pain because of the ease of access, as many with chronic pain experience daily mobility challenges.^27,28^ Indeed, one of the most significant barriers for this population is the need to travel for care.^29^ Self-directed internet-based interventions allow individuals to engage with content at their own pace and schedule, which aligns with activity pacing principles commonly used in chronic pain management.^30^ This flexibility allows individuals to pause engagement during pain or fatigue flare-ups, and resume when able. Self-directed interventions also promote autonomy and active-decision making,^31^ both of which are often undermined for those experiencing chronic pain due to feelings of powerlessness.^32^

There has been extensive work to translate effective therapist-delivered in-person psychological therapies to effective self-guided internet-based programs for chronic pain.^33^ For ACT, recent systematic reviews have identified over 20 internet-delivered ACT-based pain management programs.^23–25^ However, most require therapist support, are only available in non-English languages, or are available at a substantial cost. Therapist-supported interventions in particular require the ongoing involvement of trained professionals to support such programs, with costs needing to be borne by either the patient or the health system.^34,35^ Self-directed internet-based pain management programs without therapist involvement have also been found to be beneficial for individuals with chronic pain.^36,37^ As rates of chronic pain continue to rise, and with increasing calls for more accessible interventions and care,^3^ such programs could play a key role within stepped-care models. This may be particularly relevant in publicly funded systems like Canada, where access to publicly covered psychological pain services is limited and wait times for specialists are can be substantial. Despite this potential, ACT programs such as these remain underexamined among Canadian chronic pain populations, and specifically patients who are on waitlists to receive specialized care, despite emerging evidence suggesting that this approach can provide meaningful effects in pain-related outcomes and QOL.^38^

### Present study aims

Given the challenges in timely access to chronic pain interventions, our group of pain specialists, clinicians with ACT expertise, and patient-partners with lived experience developed an online self-directed ACT-based program utilizing evidence-based strategies found to be effective for individuals living with chronic pain. This study reports on the feasibility and pilot outcomes of the IMPACT (Internet-delivered Multidisciplinary Pain Acceptance and Commitment Therapy) program, tested with chronic pain patients on waitlists at tertiary pain clinics for specialized care, providing insight into the potential for real-world integration within publicly funded services, a population that remains under-researched. The primary objective was to assess feasibility outcomes, specifically: examining engagement rates and sample characteristics of participants, and evaluating acceptability outcomes through engagement, and user impressions of the program. Secondary objectives included evaluating pilot outcomes related to QOL, pain, and mental health, comparing pre- and post-intervention and 6-month follow-up.

## Materials and methods

This study was approved by the University of Manitoba and Shared Health Research Ethics Boards (HREB; #H2019:417). The development and feasibility study was funded by a Health Sciences Center Foundation operating grant. Patient consent was obtained prior to data collection (see below for further details).

### IMPACT program description

The IMPACT program was designed as an online, self-directed intervention based on core ACT strategies as well as supplemental evidence-based content for chronic pain populations. A manuscript describing the full development of the IMPACT program is currently in progress. Briefly, the program was collaboratively designed and implemented with patient-partners, all of whom had lived experience with chronic pain, and a multidisciplinary group of chronic pain experts, including health professionals in anesthesiology, physical and rehabilitation medicine, and clinical health psychology. Some of the patient-partners and pain experts also had experience and/or expertise with ACT. To ensure that the program was user-friendly and engaging, the team also included an educational expert in internet-delivered programming from the University of Manitoba’s Office of Innovation and Scholarship in Medical Education. The program was offered through the web-based platform *Minddistrict*, a platform that specializes in behavioral health applications. Participants were provided with their own password-protected user accounts to access the program on *Minddistrict*. The program, including homework content, could be accessed via an internet browser and included an option for a mobile app on personal devices.

The IMPACT program consists of 11 modules: an introductory module (excluded from module completion analyses), 7 core modules (6 containing standard ACT content^39^ and 1 focused on physical activity), as well as 3 supplemental modules providing evidence-based information on medication, sleep strategies, and effective communication skills. The ACT-specific core modules included learning and skills-based activities on acceptance/willingness, values, cognitive defusion, mindfulness, self-as-context (i.e., self-compassion), and committed action (see Supplemental Material A for module overviews). The information in each module was presented using multi-media and interactive components, including guided audio meditations, videos from experts, character vignettes, reflective, and/or written exercises to complete within the module, video clips featuring patient-partners, and homework (i.e., exercises to practice in between completion of modules). Further, each module had brief evaluation questions at the end to obtain participant ratings and comments on the content.

Core modules were designed to be completed in a specific order for scaffolded learning. Each module was released a week after completing the prior module and the evaluation items, to allow for skill practice opportunities. Three supplemental modules were available to access at any time, including before the core modules were accessed.

### Participants

Participants were adults (≥18 years) referred to, and on the waitlist for, specialized pain services through an interdisciplinary outpatient chronic pain clinic or behavioral services for chronic pain at the Health Sciences Center (HSC), the province’s largest tertiary and trauma hospital in Manitoba, Canada between January 1^st^, 2020, and December 31^st^, 2020. Those waiting patients were invited to participate in the study by mailed letter. The letter contained general study information and contact details for the research coordinator. Interested patients contacted the research coordinator via phone or e-mail and provided contact information. The research coordinator then contacted the patients by phone for a consent review and did a brief structured interview for eligibility screening.

Eligibility criteria included being 1) a Manitoba resident, 2) having regular access to an internet-connecting device, 3) English proficiency in reading and writing, and 4) chronic pain for at least 6 months, inclusive of all types of chronic pain (e.g., chronic pelvic pain),^40^ similar to other ACT trials in this population.^41,42^ We selected the 6-month cutoff given that some guidelines, particularly in occupational rehabilitation, focus on 6 months as the point when pain should be managed as a chronic, independent condition rather than just a symptom of a past injury (i.e., guidelines from Workplace Safety and Insurance Board of Ontario).^43^ Historically, the IASP has defined chronic pain as longer than 3–6 months. The 6-month time frame was used as a conservative chronicity definition. Exclusion criteria included a) participation in the last 6 months in psychological treatment for pain management or b) having an unmanaged or severe psychiatric condition, including having either active self-reported suicidal ideation, active psychosis, or psychiatric hospitalizations within the last 6 months. The rationale for excluding those who had recently received psychological treatment for pain management was to engage in a therapy naïve group and to mitigate the risk that prior therapy could act as a potential confounding factor given the durability of these clinical interventions. Exclusion criteria were purposefully kept minimal to best represent the population seeking care for chronic pain.

As this was a feasibility study, formal power calculations were not deemed appropriate.^44,45^ The sample size was determined pragmatically based on recruitment capacity, available resources, and timelines rather than being powered to detect efficacy outcomes. The target sample size was informed by best practice recommendations for feasibility and pilot studies.^46^

### Procedure

Informed consent was collected both verbally during the screening call and electronically via *Research Electronic Data Capture* (REDCap), a secure web-based data capture tool hosted on University of Manitoba servers.^47,48^ After providing consent, participants completed baseline clinical measures online through REDCap. Participants were then enrolled in the *Minddistrict* platform by the research coordinator and had to activate their account to access the program.

Participants could complete the program in as little as 6 weeks and otherwise had up to 3 months, with flexibility for longer time if requested and actively engaged. While the program was self-directed, participants received supportive check-in communications from the research coordinator via phone, e-mail, or direct messaging within the *Minddistrict* platform if there was no online activity for 1 month. These check-ins served both to encourage engagement and for the research coordinator to troubleshoot any technical issues that may have arisen. These did not involve clinical intervention. Participants received three e-mail reminders and one phone call before being assumed lost to follow-up. Post-program measures were collected through REDCap after program completion (3 months after enrollment), and again 6 months after that (i.e., 9 months after enrollment).

### Study measures and outcomes

#### Demographics

Demographic information was collected at baseline, including age, gender, marital status, highest education level, occupation, and income. Details of pain conditions were also collected, including duration, location, diagnosis, previous treatments, and treatment providers.

#### Primary outcomes

##### Feasibility

Feasibility was assessed in two primary ways. First, we assessed the recruitment rate for the study. Specifically, recruitment was assessed by tracking the number of people who: 1) were invited from the waiting list (sent letter), 2) expressed an interest in participating (contacted research coordinator), 3) completed screening and provided consent to participate, and 4) activated a profile in the program. *Response rate* was calculated based on the proportion of individuals who contacted the study coordinator relative to the number of participants invited to participate. *Recruitment rate* was calculated based on the proportion of individuals who consented to participate relative to the number of participants who were interested and were eligible. *Participation rate* was calculated based on the proportion of those who consented relative to those who activated the program. *Completion rate-post-measures* were calculated based on those who activated the program to those who completed the immediate post-program measures, regardless of module completion. *Completion rate-post-measures-modules* was calculated by the number of participants who activated their profile relative to those who completed both the six core modules and the post-program measures. *Completion rate-follow-up-measures-modules* were calculated at 6-month follow-up based on the number of people who activated their profile relative to those who completed the six core modules and the 6-month follow-up measures.

Second, in line with Orsmond and Cohn (2015),^49^ we examined participant characteristics of the consenting and participating sample (differentiating full from partial completion) as a measure of feasibility. Participant information was collected through a demographics questionnaire that was administered at the outset of the study. Questions included age, gender (male/female/transgender/two-spirit/other), marital relationship (married/single/common law/divorced, or separated/widowed), living location (urban/rural), education level (less than high school/high school/college or technical school/university graduate/post-graduate degree/other certificate or diploma/other education or course), work status (retired/disability/employed/unemployed), pain diagnosis (if any: osteoarthritis/degenerative disc disease/fibromyalgia/myofascial pain syndrome/migraine headaches or chronic daily headaches/trigeminal neuralgia or facial nerve pain/functional abdominal pain [e.g., irritable bowel syndrome]/gynaecological or pelvic pain/diabetic neuropathy/postherpetic neuralgia or herpes zoster [i.e., shingles]/surgical pain [pain that began only after surgery was performed in the area]/trauma pain [e.g., motor vehicle accident, physical assault, and work accident]/non-cardiac chest pain/other), and chronicity of pain including pain onset.

Acceptability was assessed via qualitative and quantitative, self-reported, and study-specific participant questions. Qualitative acceptability data were obtained at the outset of the study, and when a participant withdrew from the program. Initially, participants responded to two open-ended questions querying their interest and commitment to the program: “Why are you doing this treatment?” and “How will you make time to complete this?” in the first introductory module. When an individual withdrew from the study, a research assistant worked to contact those individuals and obtain correct reasons for withdrawal.

Additionally, quantitative participant ratings and feedback were used to assess acceptability. At the end of the IMPACT program, participants were asked questions related to the program itself, including user-friendliness and clarity (6-point Likert scale, 1 [“*not at all*”] to 6 [“*completely*”]). Participants were asked how user-friendliness could be improved (options: providing more in-depth information, improved navigation, improved layout, segmenting key information with bullet points, other), and which modules, if any, were unclear and why (options: not enough information, poorly worded, not enough examples, too much on the same screen, other). If participants did not complete the full program, they were also asked to highlight what the perceived challenges and barriers were to get through the program (options: time commitment, pain [e.g., from sitting too long, typing, etc.], readability of programs, user-friendliness, and other). Participants were asked to indicate which aspects they found most and least helpful in the modules (options: video, audio, character vignettes, metaphors, written explanations, exercises, examples, homework, and other). Furthermore, participants were asked whether they would recommend the module to others (yes/no), obtained directly through the *Minddistrict* platform. We also tracked the number of modules completed per participant to understand the overall level of engagement within the program. Participants rated, at the end of each module, the clarity and the level of helpfulness on a 5-point Likert-type scale; higher scores indicate greater clarity/helpfulness. These assessments collectively served as an evaluation of the acceptability and suitability of the intervention and study procedures, in line with recommendations.^49^

#### Secondary pilot outcomes: pain, daily functioning, and mental health

We used validated self-report measures to assess pilot outcomes at baseline, post-program, and at 6-month follow-up. Most measures were drawn from the *Patient-Reported Outcomes Measurement Information System* (PROMIS) item bank, which has been used and validated with many chronic pain populations. Each of these short-form PROMIS measures has between 4 and 10 items. We used the following measures: Pain Interference,^50,51^ Physical Function,^52^ Psychosocial Illness Impact,^53^ Fatigue,^54^ Anxiety, and Depression.^55^ Additionally, the Global Health scale was used which assesses health-related QOL across two domains: physical health [i.e., fatigue, pain, and physical function) and mental health [i.e., social health and emotional distress).^56^ All measures utilized Likert-scale response options. Higher scores for the pain interference, psychosocial illness impact, fatigue, anxiety, and depression measures are indicative of worse functioning, while the opposite is true for global health and physical functioning.

In addition, participants completed validated pain-specific measures including the *Pain Catastrophizing Scale* (PCS),^57^ the short form of the *Pain Self-Efficacy Questionnaire* (PSEQ),^58^ and the *Chronic Pain Acceptance Questionnaire* (CPAQ)^59^ The PCS is a 13-item measure, which uses a 5-point Likert-scale, ranging from 0 (“*Not at all”*) to 4 (“*All the time*”). Higher scores represent more negative thinking and appraisals about chronic pain experiences. The PSEQ uses four items to assess participants’ confidence in managing life despite pain. The participant ranks the items on a 6-point Likert-scale, ranging from 1 (“*Not at all confident”*) to 6 (“*Completely confident*”). Higher scores reflect greater pain self-efficacy. The CPAQ is an 8-item questionnaire, where participants rate items on a 7-point scale from 0 (“*Never true*”) to 6 (“*Always true*”). There are two subscales, 1) activity engagement, which measures the degree to which a person engages in life regardless of pain, and 2) pain willingness, which is described as the willingness to experience pain (e.g., not avoiding engaging in behaviors that limit contact with pain). Higher scores on the CPAQ reflect greater acceptance of chronic pain.

### Statistical and qualitative analyses

Participant characteristics as well as feasibility outcomes were examined using descriptive statistics including means, standard deviations, frequencies, and cross-tabulations. To examine potential demographic differences across program engagement, chi-square tests were conducted, comparing three groups: 1) No activation group – those who consented but did not activate the program, 2) Partial completers – those who activated the program and completed at least one but not all core program modules, and 3) Full completers – those who completed all core modules, and both the post-program and 6-month follow-up evaluations. Information on the other non-core modules completed was collected and descriptively presented. Open-ended responses to the two question participants answered at the beginning of the program, “Why are you doing this treatment?” and “How will you make time to complete this?” were analyzed independently by two research staff using qualitative content analysis.^60,61^ All disagreements were resolved through discussion and consensus. All themes were reviewed and refined by the broader research team. Representative quotes were selected to illustrate each theme.

For secondary outcomes, repeated measures of ANOVAs were conducted to evaluate pre- and post-program clinical outcome changes over time with partial completers and full completers. The assumptions of repeated measures of ANOVAs were assessed and analyzed, prior to the analysis, with partial and full completers. Normality was examined using Shapiro–Wilk tests and visual inspection of Q–Q plots, which indicated that the data were approximately normally distributed, with small minor deviations from normality which were not severe enough to violate the assumption of normality. In cases where Mauchly’s Test of Sphericity was significant (e.g., depression), a Greenhouse–Geisser correction was used. Outliers were assessed using boxplots, and only a small number of modest outliers were identified; thus, all cases were retained for analysis. Hedges’ *g* was used to estimate effect sizes, where *g* = 0.15, 0.40, and 0.75, indicate small, medium, and large effect sizes, respectively.^62^ Given the feasibility nature of this trial, missing data were an important aspect to understand and report, but no imputation methods were utilized.

We expected participants would not fully benefit from the program without completing all core ACT modules, and a pre-specified post-hoc repeated measures ANOVA was also conducted with completion status (partial versus full) included as a between-subjects factor. All quantitative analyses were conducted using SPSS 29.0.1.1.

## Results

### Recruitment and participant characteristics

**Figure 1.**
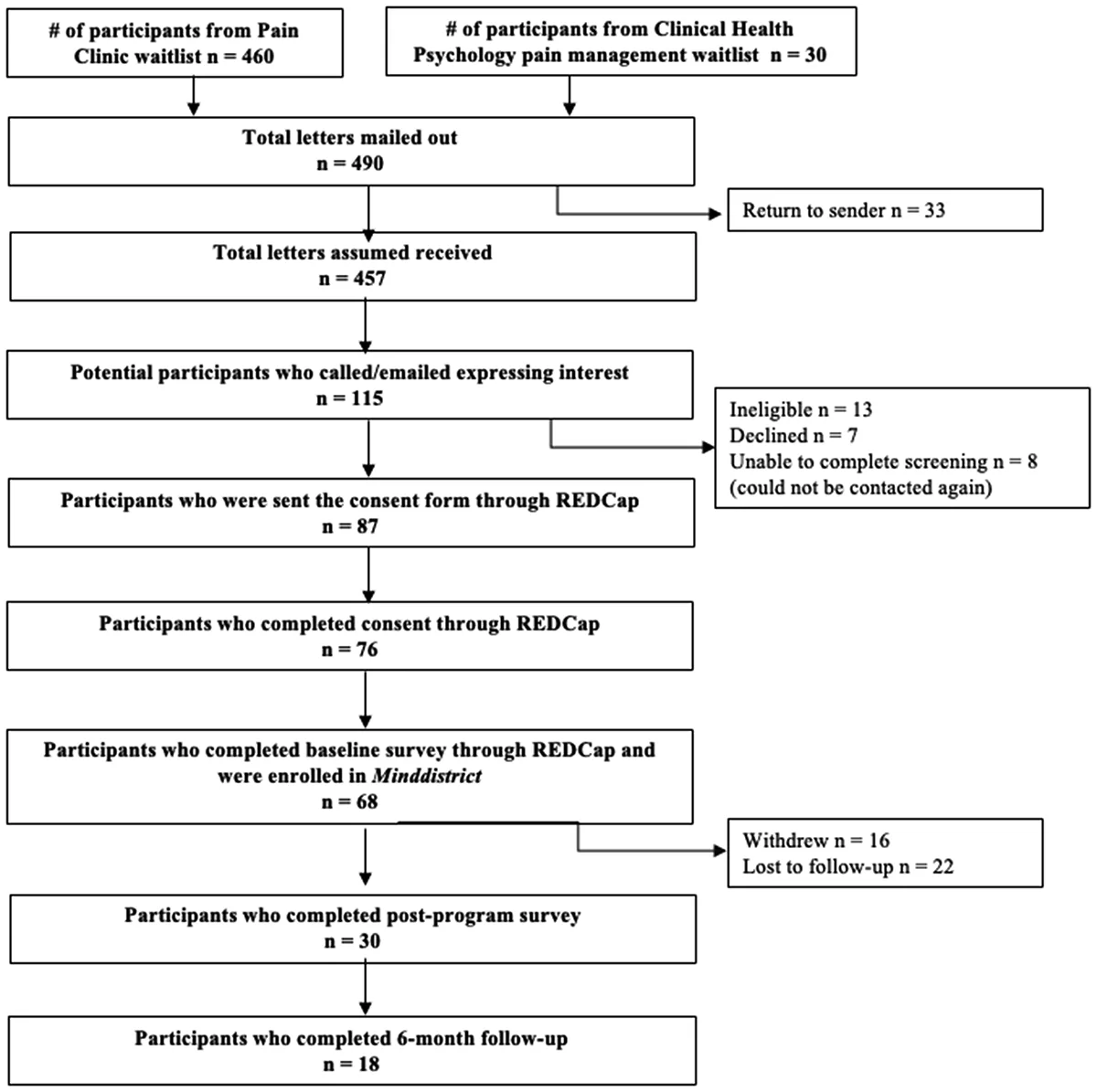
Recruitment flowchart.

Recruitment letters were mailed to 490 individuals, of which 33 were undeliverable. Of the 457 individuals who were presumed to have received letters, 115 contacted the study coordinator, yielding a response rate of 25.2% (see Figure. 1 for recruitment flowchart). Thirteen were found to be ineligible, due to no computer or internet access (*n* = 6), recent psychological treatment for pain (*n* = 3), severe depression and suicidal ideation (*n* = 3), or having moved out of province (*n* = 1). A total of 76 individuals were formally enrolled through completion of the consent form, yielding a recruitment rate of 74.5% (76/102).

To contextualize feasibility outcomes and assess what population the intervention reached, participant characteristics were examined. Enrolled participants were, on average, 54.96 years of age (Standard deviation [*SD*] = 13.83) and were living with pain for an average of 11.37 years (*SD* = 11.45). The majority were female (74.3%) and were married or in a common law relationship (64.9%). Of those enrolled individuals, 68 activated their *Minddistrict* platform profile, resulting in a participation rate of 89.5%. Detailed demographic characteristics of these participants are presented in Table 1.

**Table 1.** Demographic characteristics by completion percentage.

Characteristic	Total *N* = 68 *n* (%)	Non-completers *n* = 16 *n* (%)	Partial completers *n* = 34 *n* (%)	Full completers *n* = 18 *n* (%)	*p for F or X^2^*
Age (*M* (*SD)*, range)	54.7 (14.3), 22.4–83.0	51.1 (18.1), 22.4–78.8	54.4 (12.7), 29.9–77.8	58.4 (13.6), 38.2–83.0	.359
Gender					.305
Female	51 (75.0)	12 (75.0)	23 (67.6)	16 (88.9)	
Male	16 (23.5)	4 (25.0)	10 (29.4)	2 (11.1)	
Marital status					.239
Married or in common law relationship	47 (69.1)	11 (68.8)	22 (64.7)	14 (77.8)	
Single	13 (19.1)	5 (31.3)	7 (20.6)	1 (5.6)	
Separated, divorced or widowed	7 (10.3)	0 (0.0)	4 (11.8)	3 (16.7)	
Highest level of education					.521
≤High school diploma	16 (23.5)	4 (25.0)	8 (23.5)	4 (22.2)	
College or technical school or another certificate/diploma/other education or course	33 (48.5)	7 (43.8)	15 (44.1)	11 (61.1)	
University or post-graduate degree	18 (26.4)	5 (31.3)	10 (29.4)	3 (16.7)	
Location of residence					.094
Urban	48 (70.6)	8 (50.0)	26 (76.5)	14 (77.8)	
Rural	19 (27.9)	8 (50.0)	7 (20.6)	4 (22.2)	
Employment status					.696
Retired	21 (30.9)	5 (31.3)	8 (23.5)	8 (44.4)	
Disabled	12 (17.6)	2 (12.5)	6 (17.6)	4 (22.2)	
Employed	27 (39.7)	7 (43.8)	15 (44.1)	4 (22.2)	
Unemployed	8 (11.8)	2 (12.5)	4 (11.8)	2 (11.1)	
Pain location^ab^					
Head	21 (30.9)	4 (25.0)	10 (29.4)	7 (3.9)	.673
Shoulder(s)	27 (39.7)	5 (31.3)	16 (47.1)	6 (33.3)	.401
Neck	34 (50.0)	7 (43.8)	18 (52.9)	9 (50.0)	.776
Mid-back	24 (35.3)	7 (43.8)	11 (32.4)	6 (33.3)	.750
Lower back	50 (73.5)	13 (81.3)	22 (64.7)	15 (83.3)	.334
Ankle(s) or Foot/Feet	25 (36.8)	9 (56.3)	9 (26.5)	7 (3.9)	.143
Knee(s)	26 (38.2)	6 (33.3)	15 (44.1)	5 (27.8)	.461
Hand(s) or Wrist(s)	24 (35.3)	6 (33.3)	12 (35.3)	6 (33.3)	.964
Buttocks	20 (29.4)	4 (25.0)	7 (20.6)	9 (50.0)	.089
Multiple joints	24 (35.3)	7 (43.8)	11 (32.4)	6 (33.3)	.750
Years living with pain (*M* (*SD)*, range)	11.2 (11.7), 1.0–44.00	16.6 (13.3), 1.0–40.0	7.8 (8.9), 1.0–41.0	12.5 (13.4), 1.0–44.0	.**042**
Pain diagnosis (yes)^b^	53 (77.9)	14 (87.5)	25 (73.5)	14 (77.8)	.630
Osteoarthritis/Degenerative Disc Disease	25 (36.8)	8 (50.0)	11 (32.4)	8 (44.4)	.662
Fibromyalgia	12 (17.9)	6 (33.3)	7 (20.6)	1 (5.6)	.249
Migraines/Chronic Daily Headaches	11 (16.2)	3 (18.8)	5 (14.7)	4 (22.2)	.675
Post-Trauma Pain (e.g., motor accident)	11 (16.2)	7 (43.8)	1 (2.9)	6 (33.3)	.**007**
Professionals visited for pain^ab^					
General practitioner/family doctor	64 (94.1)	14 (87.5)	32 (94.1)	18 (100.0)	.182
Acupuncturist	23 (33.8)	5 (31.3)	15 (44.1)	3 (16.7)	.112
Rehabilitation/Physical Medicine	23 (33.8)	3 (18.8)	14 (41.2)	6 (33.3)	.261
Neurologist	17 (25.0)	4 (25.0)	8 (23.5)	5 (27.8)	.962
Chiropractor	38 (55.9)	10 (62.5)	18 (52.9)	10 (55.6)	.864
Physiotherapist	35 (51.5)	9 (56.3)	15 (44.1)	11 (61.1)	.527
Methods used for pain^ab^					
TENS	33 (48.5)	9 (56.3)	14 (41.2)	10 (55.6)	.545
Acupuncture	29 (42.6)	6 (33.3)	17 (50.0)	6 (33.3)	.396
Manipulation	21 (30.9)	4 (25.0)	11 (32.4)	6 (33.3)	.822
Heat therapy	34 (50.0)	5 (31.3)	17 (50.0)	12 (66.7)	.118
Bed rest	23 (33.8)	5 (31.3)	11 (32.4)	7 (3.9)	.883
Massage therapy	40 (58.8)	8 (50.0)	21 (61.8)	11 (61.1)	.653
Ultrasound	16 (23.5)	5 (31.3)	5 (14.7)	6 (33.3)	.253
Exercise	44 (64.7)	11 (68.8)	21 (61.8)	12 (66.7)	.934
Supplementary modules completed					
Medication	48 (70.6)	0	31 (91.2)	18 (100.0)	–
Sleep	42 (61.8)	0	24 (70.6)	18 (100.0)	–
Communication and relationships	32 (47.1)	0	14 (41.2)	18 (100.0)	–
Planned to set-aside time					
Yes	25 (51.0)	–	16 (47.1)	9 (50.0)	.823
Surgery for pain					
Yes	18 (26.5)	7 (43.8)	7 (20.6)	4 (22.2)	.217

*Note.* Bolded indicates significance. ^a^Participants could select multiple answers. ^b^The options only represent the top responses.

Of those that activated their *Minddistrict* profile to access the program, 52 participants (76.5%) completed at least 1 program module, with an average of 3.09 (*SD* = 3.13; range: 0–7) core modules completed. As the three supplemental modules were accessible at any time, an average of 6.44 (*SD* = 3.82; range: 1–11) total modules were completed, including both core and supplemental. Thirty participants completed the post-program survey, resulting in a post-study completion rate of 44.1%, though not all of these individuals completed every module, or any. Eighteen participants (26.5%) completed all the core program modules and the post-program survey and 17 participants (25.0%) completed the 6-month follow-up. Among those that completed at least one core module, supplemental module completion rates were 94.2% (*n* = 49), 80.8% (*n* = 42), and 61.5% (*n* = 32) for the medication, sleep, and communication modules, respectively. Participant characteristics stratified by the level of engagement (i.e., those who did not activate, partial completers, and full completers) are also presented in Table 1 to demonstrate the resulting sample characteristics across these subgroups.

#### Differences between completers, partial completers, and non-completers

There were two significant differences between full completers, partial completers, and non-completers in terms of pain diagnoses and years living with pain. Those who did not complete any modules appeared to have been living with pain for longer (*M* = 16.6, *SD* = 13.3) compared to partial (*M* = 7.8, *SD* = 8.9) and full completers (*M* = 12.5, *SD* = 13.4; *F*(2, 64) = 3.33, *p* <.05). Additionally, post-trauma pain was more commonly diagnosed in non-completers (*n* = 7, 43.8%) and full completers (*n* = 6, 33.3%), as compared to partial completers (*n* = 1, 2.9%).

#### Reasons for interest in the program

Four primary themes were identified from responses to the questions of “why are you doing this treatment?” (see Table 2 for illustrative quotes per theme). The first theme was the desire to *reclaim life*. Within this theme, participants highlighted goals to improve various aspects of their life, such as QOL, enjoyment of life, increasing mindfulness and acceptance, and regain emotional control of their life. The second theme was the *negative impacts of chronic pain*. Participants indicated they wanted to learn how to reduce their pain and learn pain reduction techniques and strategies. The third theme was *seeking improved health*, where participants reported wanting to improve their general wellness and health, including becoming more active and adopting better habits. Finally, the fourth theme was *engagement and accessing resources*, where participants reported joining the program to access alternate resources while on the waitlist for pain services.

**Table 2:** Representative Quotes for “Why are you doing this treatment?”

Theme	Definition	Representative Quotes
“I want my life back”	Wanting to improve various aspects of their life to lead to better quality of life, enjoyment, self-improvement, acceptance, and to regain/reclaim control of their lives.	“To help myself live with my pain and the changes in my body since my Injury. I’m afraid I’m losing too much of myself to my new limitations, and *hope this will help me lessen how much I feel I’m losing*” “I am doing this to see if it can have a positive impact on my life. *My goal would be to have a more positive, hopeful outlook for the future* and maybe have more energy.” “I am currently on a wait list for the Pain Clinic and have been living with chronic since my surgery in 2019. I want to get off my medication and *have a better quality of life with my kids*.” “*I want to take charge of my life and not pain taking over my life*. I know that there are days that are bad and I feel bad as well. Now i just need to listen to my body, if its hurting then i need to slow down and rest and not to feel bad about it, and just look forward to the days that ’’m my old self again”“ “”*I want my life back*. I spend so much time trying to deal with my pain that there isn’t much energy left over. I still try to do things but they have to be things I can do sitting down as standing or walking isn’t possible for longer than 10 min. I still draw and paint and sew but I used to be so much more active. I would love to go dancing again at some point.“” “”I hope to start living and not feel like I am just surviving. I feel like I am already missing so much”” “”I would like to get back to a positive outlook towards and during daily function. My goals would be set with simplicity and self awareness that I hope to gather from this program”” “”*I yearn to having a purpose, something to do every day*. I look forward to the program like its my job. I want to stop feeling guilty about what I ca’’t do. I want to stop feeling like I have no right to feel bad because there are a lot of people worse off than me. I want to stop hating myself and to not feel like a loser. Generally, I want a better feeling about myself as a whole-both physically and mentally.“” “My goal is to reclaim the parts of my life that pain has taken from me.”
Negative impacts of chronic pain	Wanting to gain a sense of control over the pain they experience to and manage their pain and learn pain reduction strategies and techniques, due to the exhaustion and physical limitations of dealing with chronic pain.“”To stop my pai””	“”I am doing this program to try and gain some more control over my pains”” “”so how did these people get cured? wha’’s the answer”” “”’’m looking for coping skills when the pain sets in. Ideally find a way to get pain relief but the nerve damage cannot be reversed”” “”I am doing this because I will try anything that may help my pain.“” “”Learn more about pain management/living with chronic pain”” “”Learn new ways manage pai”” “”*I will do anything to reduce my pain* and i’’s impact on my life. My goals are to develop more tools to cope with my pain. I want to feel supported and have access to resources”” “”I have been working hard to help my pain and stop from losing my life to it. *I hope by finishing this program some of my daily pain will ease*””
Seeking improved health	Wanting general wellness or improved health in their life to gain more strength and energy, be more active, and have stable health and wellness habits.“”I am doing this to see if it can have a positive impact on my life. My goal would be to have a more positive, hopeful outlook for the future and *maybe have more energy*””	“”I am doing this to learn how to cope with chronic pain so I am able to help myself to do more of the things that I enjoyed in the past. My pain isn’t as bad as some of the people in the previous video, *so my goal is to become more active and enjoy life more again*”” “”’’m doing this out of curiosity. ’’m tired of being in pain, not able to walk my dog. The pain is noe complicated by cataracts. I feel like ’’m just putting in time until I die. *My goal is to become active again*”” “”I want my life back. I spend so much time trying to deal with my pain that there isn’t much energy left over. I still try to do things but they have to be things I can do sitting down as standing or walking isn’t possible for longer than 10 min. I still draw and paint and sew but I used to be so much more active. *I would love to go dancing again at some point.“*“ “”Self-management is my best option for being able to improve and maybe even recover from my illness. I want to be as active as possible in my own wellness. My goals are *to create solid wellness habits* and for my day-to-day experience with my health to become as stable as possible””
Engagement and accessing resources	Wanting to engage in and access alternative resources that may be helpful such as the chronic pain waitlist, but receiving support and resource while they are waiting.	“”I am currently on a wait list for the Pain Clinic and have been living with chronic since my surgery in 2019. I want to get off my medication and have a better quality of life with my kids”” “”I’m doing this program because I’m waiting to get into the pain management clinic. When I was asked to participate, *I thought why not. If it works great, if not I tried*.“” “”I will do anything to reduce my pain and i’’s impact on my life. My goals are to develop more tools to cope with my pain. *I want to feel supported and have access to resources”” *

Note. Several quotes fell into multiple themes. These only represent some but not all of the quotes.

#### Commitment to the program

For the question “How will you make time to complete this?,” there were two main response themes. Some participants reported specific strategies, such as setting time aside or using reminders to stay on track (*n* = 25, for example, “schedule the time and use reminders,” “Get up earlier to focus on it before work. Try to make time for it before bed as well,” “I have time to do it while my kids are at school or in the evening when they are gone to bed”). Others noted having no set plan in place (*n* = 24), expressing that they would try and make time (e.g., “I will make time because I want to get back to the tasks I enjoyed.,” “I just take every day a day at a time. I’m a busy woman and was told I sound [should] be able to fit this into my schedule so I’m doing my best to try,” “If I want to learn different techniques an [and] skills to lessen my daily pain, I must make time. That’s all on me, no body can help me if I don’t try an [and] help my self first.”).

#### Reasons for withdrawal

Participants who subsequently did not complete all modules and/or post-program measures provided various reasons for no longer wanting to participate in the program. The most common barrier was the program’s time commitment required. Additionally, some participants faced technological barriers. Others expressed concern that the program did not meet their expectations, such as looking for pain relief rather than pain management. Few participants misunderstood the focus of the program, believing it was for people whose pain was caused by psychological factors, rather than physical factors. Finally, others found the end of module questionnaires onerous and repetitive to answer.

#### Program feedback

Most participants who completed the post-program questionnaires rated the program as either “*mostly*” (8/25, 32.0%) or “*completely*” user-friendly (12/25, 48.0%). The most common suggestions for improvement were to segment key information with bullet points (*n* = 12), provide more in-depth information (*n* = 7), improve system navigation (*n* = 7), or other (*n* = 6, e.g., improve program glitches, opportunity to skip modules). Similarly, most participants found the program instructions to be “*mostly*” (10/25, 40.0%) or “*completely*” clear (13/25, 52.0%). Among those that did not find all modules completely clear (12/25, 48.0%), the majority identified the values (8/12, 66.7%) and the thoughts and self-compassion modules (6/12, 50.0%, each) as more challenging to follow. These individuals identified aspects such as not having enough examples (e.g., how patient-partners described doing mindfulness; 8/12, 66.7%), too much information being on the same screen (7/12, 58.3%), not enough information (5/12, 41.7%), or other reasons (3/12, 25.0%, e.g., tasks were overly complex).

When asked which elements were the most helpful about the modules, participants cited that the written explanations (*n* = 16, 61.5%) and the examples (*n* = 16, 61.5%) were the most helpful, followed by the videos (*n* = 13, 50.0%) and the exercises (*n* = 13; 50.0%). In contrast, aspects seen to be the least helpful were the metaphors (*n* = 8, 30.8%) and the audio (*n* = 7, 26.9%).

All participants who completed the self-as-context/self-compassion and the committed action module said that they would recommend it (*n* = 18 each), while the least recommended module was the acceptance/willingness module (*n* = 24/32 recommended, 75.0%). Clarity varied across modules, with means ranging from 3.53 to 4.41 (maximum 5 = greater clarity). The least clear module was the acceptance module (range 2–5), while the clearest module was exercise (range 4–5). Helpfulness varied as well, with means ranging from 3.50 to 4.28 (maximum 5 = greater helpfulness), the least helpful being the medication (range 1–5) and the thoughts (range 1–5) modules. The most helpful was the sleep module (range 2–5).

### Secondary pilot outcomes

**Figure 2.**
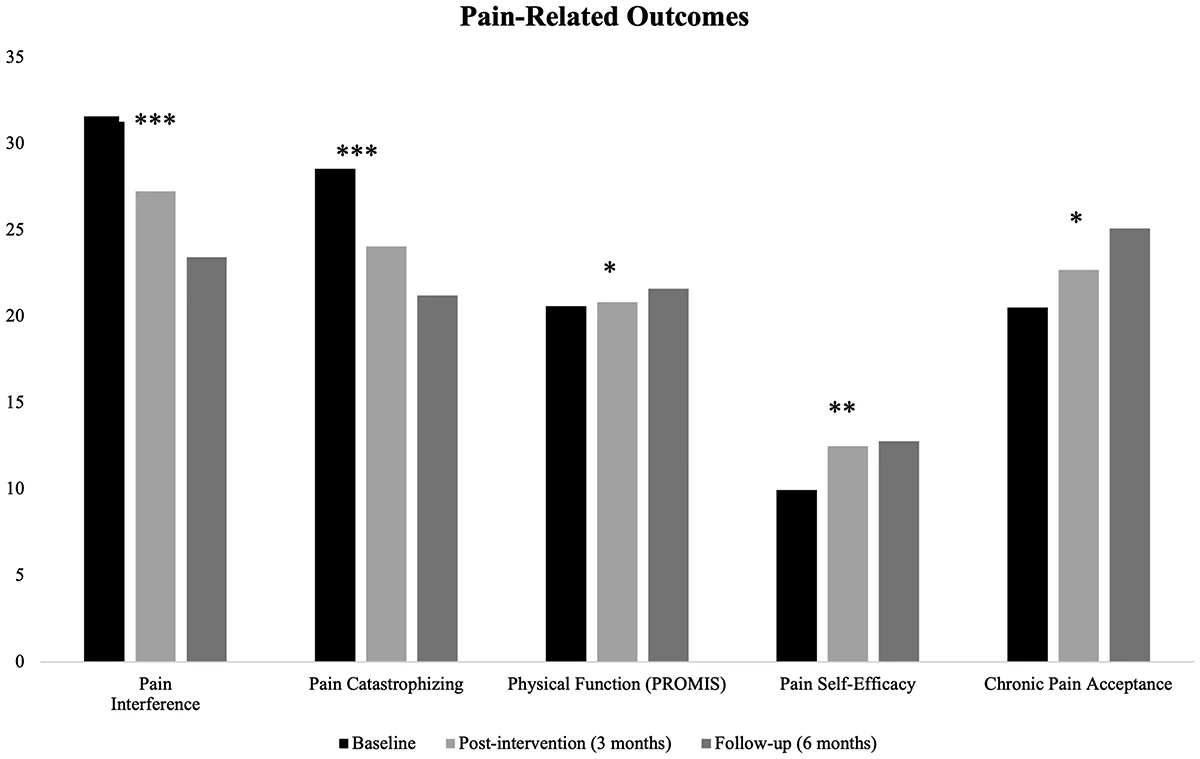
Pain-related outcomes over time for full completers and partial completers.

Note. n = 30. For pain interference and catastrophizing, higher scores represent worse outcomes, while for pain self-efficacy, physical functioning, and chronic pain acceptance, higher scores represent better outcomes. *p < .05, **p < .01, ***p < .001.

**Figure 3.**
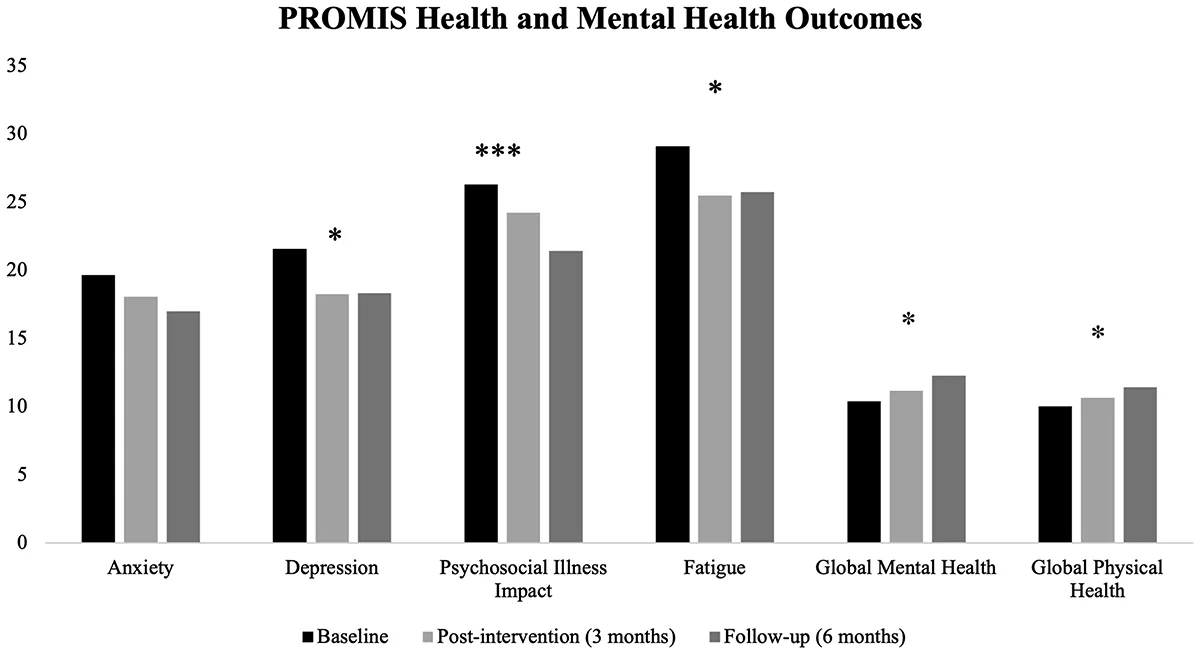
Health and mental health-related outcomes over time for completers and partial completers.

Note. n = 30 For anxiety, depression, psychosocial illness impact, and fatigue, higher scores represent worse outcomes, while for global mental health and global physical health, higher scores represent better outcomes. *p < .05, **p < .01, ***p < .001.

Comparing secondary outcomes over time from baseline to the 6-month follow-up, pain interference (*F*(2) = 10.65, *p < *.001, *g* = −0.92), pain catastrophizing (*F*(2) = 11.26, *p < *.001, *g* = −0.95), psychosocial illness impact (*F*(2) = 9.42, *p < *.001, *g* = −1.06), fatigue (*F*(2) = 4.23, *p < *.05, *g* = −0.41), and depression (*F*(1.40) = 5.12, *p < *.05, *g* = −0.50) all significantly decreased for partial and full completers. Physical functioning (*F*(2) = 3.77, *p < *.05, *g* = 0.68), overall global health (*F*(2) = 4.45, *p < *.05, *g* = 0.56), global health – physical (*F*(2) = 3.47, *p < *.05, *g* = 0.50), global health – mental (*F*(2) = 4.73, *p < *.05, *g* = 0.58), pain self-efficacy (*F*(2) = 7.87, *p < *.01, *g* = 0.56), chronic pain acceptance total score (*F*(2) = 4.88, *p < *.05, *g* = 0.58), chronic pain acceptance activity engagement subscale (*F*(2) = 3.95, *p < *.05, *g* = 0.54), and the chronic pain acceptance pain willingness subscale (*F*(2) = 3.70, *p < *.05, *g* = 0.51) all significantly improved across time for partial and full completers (see Figures 2 and 3, and Table 3 for means and SDs). Anxiety was the only variable that did not significantly change across time (*F*(2) = 0.75, *p = *.48, *g* = −0.26).

**Table 3.** Means and Standard Deviations of Pilot Outcomes at Baseline, Program-End, and Program Follow-up Among Partial and Full-Completers.

Characteristic	*Baseline (T1)* *Raw score: M (SD)* T-score: *M (SD)*	*T2* *Raw score: M (SD)* T-score: *M (SD)*	*T3* *Raw score: M (SD)* T-score: *M (SD)*	*Hedges g T1 to T2*	*Hedges g T1 to T3*
Pain catastrophizing	29.12 (12.98)	23.33 (17.55)	21.22 (14.76)	-0.70	-0.95
Pain self-efficacy	10.12 (5.93)	13.04 (7.12)	12.78 (6.94)	0.64	0.56
Chronic pain acceptance – total	20.44 (8.27)	22.67 (9.51)	25.11 (9.54)	0.35	0.58
Chronic pain acceptance – pain willingness scale	8.15 (4.07)	8.52 (4.80)	10.78 (6.15)	0.26	0.51
Chronic pain acceptance – activity engagement scale	12.29 (6.02)	14.15 (6.68)	14.33 (6.13)	0.31	0.54
**PROMIS Measures**					
Pain interference	31.58 (6.94) 67.68 (6.26)	27.26 (8.07) 64.27 (6.67)	23.44 (8.89) 60.96 (6.51)	-0.51	-0.92
Psychosocial illness impact	26.33 (8.89) 62.11 (9.36)	24.26 (10.09) 61.28 (10.46)	21.44 (9.80) 57.79 (10.92)	-0.32	-1.06
Physical functioning	20.60 (7.37) 35.38 (6.14)	20.85 (8.26) 35.18 (7.20)	21.61 (6.82) 36.27 (5.03)	0.17	0.68
Fatigue	29.19 (8.13) 63.20 (9.27)	25.52 (8.47) 59.29 (9.36)	25.78 (8.50) 59.74 (9.62)	- 0.62	-0.41
Depression	21.60 (9.58) 59.41 (10.94)	18.26 (9.64) 55.19 (11.59)	18.33 (8.75) 54.65 (11.29)	-0.52	-0.50
Anxiety	19.67 (6.98) 59.48 (9.89)	18.07 (7.79) 57.33 (10.94)	17.00 (7.04) 55.22 (10.63)	-0.25	-0.26
Global health	25.17 (7.41)	26.81 (8.45)	29.06 (8.79)	0.30	0.56
Global health - mental Global health - physical	10.40 (3.58) 39.24 (9.44) 10.04 (2.86) 34.61 (7.70)	11.19 (4.01) 41.28 (10.37) 10.67 (3.25) 36.25 (8.83)	12.28 (4.11) 44.09 (10.70) 11.44 (3.01) 38.49 (8.00)	0.31 0.22	0.58 0.50

*Note. N* = 27 (T2), *N* = 18 (T3). T-scores only available for PROMIS scales.

When comparing the outcomes for partial versus full completion, no significant differences were observed for anxiety (*F*(2) = 0.50, *p* > .05), the global health – physical (*F*(2) = 0.13, *p* > .05), pain catastrophizing (*F*(2) = 0.12, *p* > .05), and overall global health (*F*(2) = 0.04, *p* > .05) across time. Similarly, while pain interference (*F*(2) = 1.48, *p* > .05), psychosocial illness impact (*F*(2) = 0.17, *p* > .05), fatigue (*F*(2) = 1.27, *p* > . 05), physical function (*F*(2) = 0.71, *p* > .05) the global health – mental (*F*(2) = 0.27, *p* > .05), and depression (*F*(1.41) = 0.29, *p* > .05) did not significantly differ across time between partial and full completers, full completers tended to have higher improvement scores.

## Discussion

Chronic pain is a highly prevalent disease,^3,4^ and access to timely evidence-based pain management has historically been limited.^7,13^ The IMPACT program is an ACT-based, self-guided internet treatment developed to help increase access to evidence-based care for adults living with chronic pain. The results demonstrated the feasibility of the program for adults awaiting tertiary pain care services, an understudied population. Importantly, this study tests a self-directed online ACT program in a Canadian context, where access to publicly funded psychological services is limited and long wait times for specialized services are common. Our recruitment and participation rates were on par with other e-interventions.^63,64^ Multi-method data highlighted that, among those who provided feedback, participants found that the IMPACT program was clear and easy to use, and program content was appropriate and helpful in learning new ways to live with chronic pain. Specific suggestions for improvement are related largely to visual aspects of the program for further enhancement. Pilot outcomes provided support for improvements in pain catastrophizing, pain interference, psychosocial illness impact, fatigue, and depression, with medium-to-large effect sizes. Outcome changes were observed for both partial and full program completers from pre- to post-treatment and at 6-month follow-up. These trends for improvement align with prior research that self-guided eHealth treatments can show significant, but small effects in the short- and intermediate-term on pain-related outcomes, mental health, and self-efficacy.^38^ However, importantly, these results represent pilot trends as the study was not adequately powered to detect clinically significant findings.

The response rate of 25.2% for the current study was fairly typical for this type of intervention (i.e., online, self-directed).^65^ and further, over three-quarters of those that expressed interest ultimately proceeded, completing on average over 6 of the modules. This suggests the program could be broadly accessible and impactful when scaled up, noting that one-quarter of adults in our sample referred for specialized chronic pain services were able to access evidenced-based care instead of simply continuing to wait for treatment. This low resource program (i.e., no ongoing oversight from clinicians) was deemed acceptable and beneficial to those who participated. This has meaningful implications in Canada for the backlog of pain services that were observed post-COVID-19 pandemic.^66^

Our results suggest that the IMPACT program has the potential to be integrated within multidisciplinary pain clinics as a low-intensity treatment option within a stepped-care framework. In fact, many pain clinics across Canada have been moving toward stepped-care frameworks, which match the intensity of care to the severity and complexity of the patient’s needs.^67^ Internet-delivered programs represent a scalable, low-intensity but broad-reach interventions that can reduce clinician burden. They are being increasingly incorporated into routine care in multiple countries. Other Canadian and Australian studies have demonstrated promising findings across clinical outcomes, engagement, and adherence.^68–70^ Although the current study did not evaluate implementation into routine care, the findings related to feasibility, acceptability, and engagement add to this growing literature supporting the potential role of integrating internet-delivered programs into stepped-care clinical approaches.^68–70^ Ultimately, the integration and implementation of low-intensity programs like the IMPACT program can help increase access to evidence-based care at a much earlier time in a patient’s chronic pain journey, and have the potential to lead to meaningful changes in pain and mental health-related outcomes.

Similar to other internet-based interventions,^63,64^ we observed relatively high attrition over the course of the program, with, for example, half of participants starting the program but not completing all the core ACT modules. Reasons for withdrawal were not surprising and could be proactively addressed in future studies or clinical applications. For example, most identified time commitment as a barrier to participation. Future iterations may very likely benefit from shorter and more streamlined modules, as well as flexible content format modifications that better adapt to busy lifestyles (e.g., video or podcast-based options). Time commitment could also be addressed through intentional enhancement of motivation.^71,72^ One way we had sought to enhance engagement was through fostering participant’s reasons for engagement at the outset of the program (i.e., “*Why are you doing this*” and “*How will you make time*”). Given we still experienced attrition despite initial prompting, it is possible that the program would benefit from additional ways to enhance motivation and reduce barriers. For example, program staff could connect with participants through brief phone calls or text messages to identify treatment goals and brainstorm solutions to any barriers. This is emerging as a newer research area exploring the difference in participation in guided versus unguided online programs.^73^ In one study, individuals receiving a brief telephone-delivered motivational interviewing (MI) intervention were significantly more likely to complete the CBT program than those who did not receive the MI intervention.^74^ This suggests that integrating MI may be an effective option to integrate in future trials. That being said, the range of intensity of clinician involvement could also be considered, particularly as we consider the scalability of programs, such as the IMPACT program. Indeed, prior programs with minimal therapist contact and tailored messages, such as periodic telephone calls or e-mails,^75,76^ have been associated with lower dropout rates.^77–79^ A direction for future research may involve exploring the use of MI to help with treatment engagement and reduce dropouts both via initial telephone calls and via asynchronous methods (i.e., e-mails). Although these approaches may play a role, dropout may also be related to an extraneous variable. For example, 43.8% of the non-completers had surgery for pain compared to around 20% of the partial and full completers. It is possible that these pain-related characteristics are playing an important role in completion. Future research should explore these and other potential confounding factors through matched groups.

The level of engagement with the IMPACT program varied. Participants more frequently completed supplemental modules on medication, sleep, and communication skills than the core ACT modules. A possible reason may have been because these modules were open to access at any time. Another possible reason may have been because they were of particular interest to the participants. In fact, these modules were added based on patient-partner feedback, which may have enhanced their perceived relevance and contributed to higher engagement from study patients as well, underscoring the importance of continued integration of patient partners throughout development and testing. There may be initial enthusiasm and motivation to be involved in the program, leading participants to access all available content initially, where motivation may diminish over time. Interestingly, participants shared that some program modules were more challenging to follow compared to others, with the values module being frequently identified as one of the most difficult to follow. This result is notable considering the importance of values within an ACT framework. In fact, values are often discussed to enhance motivation by helping individuals connect to the underlying reason for engaging in particular actions.^80^ In this study, difficulty following and understanding the values-based content may have influenced participants’ experience with the program, leading them to withdraw from the study. It would be worthwhile to reexamine the values module to identify ways to enhance clarity, either by including more written explanations, examples, and exercises, as these were identified by participants as the most useful modality for presenting information. Alternatively, while the multidisciplinary team structured the core ACT modules to be delivered in a particular order, it may be possible that participants would have benefitted from more flexibility in how and when they accessed the various models. There is some evidence that this more flexible approach may enhance engagement.^67^ Nevertheless, participants who engaged with the IMPACT program described it as useful and something they would recommend to others. Future work should gather more in-depth feedback on the barriers to completion and obtain participants’ perceptions on how to increase engagement.

While not the primary aim of this study, participation in the IMPACT program was associated with several changes in pain- and health-related outcomes. In fact, both full and partial completers experienced improvements in aspects, such as pain interference, pain catastrophizing, depression, fatigue, and pain acceptance. We found that full completers tended to experience greater improvement compared to partial completers, though sample sizes for both were small due to attrition. These results suggest that a positive impact can follow from a low resource treatment option, especially since full completers had a longer pain history (average of 12.5 years) compared to partial completers (average of 7.8 years). The IMPACT program was designed to be an offered service to adults earlier in their pain management journey, which in the current project was while they were awaiting specialized pain services. This allows for the implementation of behavioral health strategies aimed at enhancing their quality of life sooner and promoting an active approach to pain management. This is in contrast to passively waiting for specialized pain services, which when received may not meet patients’ expectations.^81^ The Canadian Pain Task Force has clearly outlined the need for people to have access to timely pain care,^3^ with specific mention of the value of virtual, self-management treatment. The IMPACT program could help in this regard.

Contrary to the hypothesis that participants would only benefit by completing the entire program, partial completers also demonstrated improvements across several outcomes, suggesting that even partial use of this program may be beneficial to participants. However, comparisons between partial and full completers should be interpreted cautiously, due to the small subgroup sample sizes, missing data, and attrition, which limited the ability to detect meaningful group differences. Nevertheless, on average, partial users completed at least six full program modules and an average of three core modules, which may represent a sufficient therapeutic ‘dose.’ The identification of the lowest effective dose of ACT, as well as the specific modules that may be most critical to achieving meaningful outcomes, remains an important area for future research. It is also possible that differences in characteristics across groups accounted for these findings, such as the fact that full completers had on average close to 5 additional years with their pain condition. Future research should aim to replicate these findings with larger sample sizes and an appropriate control group.

### Strengths and limitations

This study has several strengths. First, it evaluated an internet-based, self-directed evidence-based ACT program, which addresses significant barriers frequently cited by individuals with chronic pain, such as transportation issues and scheduling conflicts.^29^ Additionally, this self-directed format did not utilize therapist support, making it more sustainable from a resource perspective, and nevertheless was deemed acceptable by participants, and had positive symptom and functioning outcomes. Thus, it has the potential to be a scalable intervention which could significantly increase accessibility for a wide range of individuals. This is particularly relevant in Canada, given the well-documented long wait times for specialized chronic pain services.^7^ Another notable strength is that the program was co-developed with patient partners, a best practice in intervention design that has been shown to enhance participant acceptability,^82,83^ improve feasibility metrics,^82–85^ and support health outcomes.^84^ Furthermore, the current study offers practical insights for the development of future internet-based programs for other researchers. For example, within our study, participant feedback suggested that examples and written explanations were the most helpful, while audio files or metaphors were less useful to participants. Similarly, high engagement with the supplementary medication module suggests that these are important topics for this population. Finally, participants’ qualitative feedback identified multiple motivating factors for participation in the IMPACT program, including reclaiming their life and improving their health. These data are unique assets to this study.

This study should, however, be considered in the context of some limitations. First, the attrition rate, while similar to other studies of this nature,^86^ may reflect a potential self-selection bias, limiting the generalizability of the findings to the broader chronic pain population in Canada. We did not conduct imputation methods for missing data in light of the feasibility focus, but this will be considered in future clinical trials. Additionally, recruitment was conducted via mailed letters, and no data were able to be collected from non-responders, making it undeterminable if there were any systematic differences between responders and non-responders. The current study was conducted primarily to assess the feasibility and acceptability of the IMPACT program, and accordingly, it was not conducted as a randomized controlled trial. It would be important for future research to examine the efficacy of the IMPACT program on pain and pain-related outcomes compared to a treatment-as-usual (i.e., waitlist control) group or another available behavioral interventions, to help solidify our understanding of the clinical utility and effect size of this online treatment program. Further, as this was considered a feasibility and pilot study, it was not prospectively registered nor had the protocol been published, which may limit transparency. Outcomes used for feasibility and acceptability were study specific, guided by prior published guidance, but may have benefitted from following established guidelines, such as those from. Outcomes used for feasibility and acceptability were study specific, guided by prior published guidance, but may have benefitted from following established guidelines, such as those from Bowen et al.^87^ While our study did not follow this guide, conceptually our outcomes are similar to Bowen’s focus on acceptability and demand. Additionally, we were not able to track specific program engagement metrics, such as how long (i.e., hours) it took participants to complete each module. The sample size, though appropriate and robust for feasibility and pilot studies,^44^ remains small, limiting the statistical power to detect the true effect of the intervention. Similarly, the convenience sample used does limit generalizability and likely has some biases for those on the waitlist who chose to participate; findings should be interpreted with caution. Integration of this type of programming outside of a research context and into a clinical context may yield a more heterogeneous group of individuals waiting for services. Finally, subgroup analyses between the three groups (i.e., full-, partial-, and non-completers) should be interpreted cautiously due to the small subgroup sample sizes. These only represent trends.

## Conclusion

This study evaluated an internet-delivered and self-directed ACT-based intervention for individuals with chronic pain on the waitlist for specialized pain management services in Canada, addressing an important gap in accessible low resource, scalable interventions for individuals awaiting specialized pain services. Findings suggest that the intervention was feasible and shows emerging acceptability and improvements in pain-related outcomes and other important health-related outcomes (e.g., QOL, depression). This internet-based ACT intervention, in addition to showing trends with promising health-related benefits, addresses several barriers commonly faced by individuals with chronic pain, such as mobility difficulties, transportation, and costs. Excitingly, Canadians can access a free online portal funded by Health Canada, with various evidence-based chronic pain resources, called the Power Over Pain Portal; the IMPACT program is currently available at no cost through this portal. Participants’ recommendations for enhancing the program from this study have been integrated into the current program. Programs like the IMPACT program are an important part of stepped-care, allowing more Canadians to access chronic pain self-management resources, with the potential to improve the lives of individuals and decrease the burden of chronic pain in Canada.

## Supplementary Material

Supplemental Material
