# Supplementary material for: Evaluation of an Internet-Delivered Multidisciplinary Pain Acceptance and Commitment Therapy (IMPACT) Program for Individuals Living with Chronic Pain: A Feasibility and Pilot Study
Source: Can J Pain. 2026 Aug 18;10(1):2696248. doi: 10.1080/24740527.2026.2696248 (PMC13488465; doi:10.1080/24740527.2026.2696248)
Supplement: Supplementary file 1 [file UCJP_A_2696248_SM3327.docx]

Supplemental Material

**IMPACT Core Modules**

| Modules | Information presented in modules |
| --- | --- |
| Welcome | Program introduction  Introduce four composite patients who will be completing the program (peer modelling) Initial People with lived experience videos |
| CORE: Willingness | Difference between external events (can be controlled/ problem solved) and internal events (control efforts often amplify)  Complete “what hurts and what do you do to get away from what hurts” exercise  Willingness metaphors (e.g., tug of war with pain monster)  Clean and dirty discomfort  “Acceptance of Thoughts and Feelings” mindfulness exercise |
| CORE: Values | What values are and the difference between values and goals  The 80^th^ Birthday Exercise  Complete “what matters to you” exercise  Complete the Values Clarification and the Values Compass exercises  “Mindfulness of the Breath” mindfulness exercise |
| CORE: Thoughts are thoughts, nothing more nothing less | Introduce the concept that our mind doesn’t always “get things right”  The “lemons lemons lemons” (Titchener’s Repetition) exercise  The “don’t think of a yellow jeep” exercise  Cognitive defusion strategies, including “Getting off your but’s” and “Having a thought versus buying a thought”.  The “Catch and Release Metaphor”  “Leaves on a Stream” mindfulness exercise |
| Exercise | Introduction to Pacing  The “Twin Peaks” model  Goal oriented pacing / activity – graded exposure  Information about the benefits, types, and intensities of exercise progression, hurt versus harm.  Diary examples to guide goal setting and track progress |
| CORE: Mindfulness | The difference between being on autopilot and being in the present moment  The “Notice Five Things” mindfulness exercise  Mindfulness of Everyday Activities  Introduction to the “Observer Self”  The “Sky” Metaphor  “Short body scan” mindfulness exercise |
| CORE: Self-Compassion | Information on what Self-Compassion is and what it is not  Compassion toward others exercise  Compassion toward our bodies  Compassionate imagery exercise  The “Compassionate Hand” exercise  The “Evaluation of Self versus Evaluation of Behaviour” Exercise  “Loving Kindness” mindfulness exercise |
| CORE: Living a Valued Life | The “Unwanted Party Guest” ACT metaphor video  Introduction to the “Willingness Switch”  The “Trying Pen” exercise  The “Passengers on the Bus” ACT metaphor video  Values Based Goal Setting and the Values Action Plan  “Path up the Mountain” mindfulness exercise |
| SUPPLEMENTAL: Communication and Relationships | Assertive communication  WIN statements and exercises  Advanced assertive communication strategies (Broken record, fogging)  Barriers to effective communication  Tips to improving relationships and support and decreasing interpersonal stress  Active listening |
| SUPPLEMENTAL: Medication | Types of pain  Goals of medication  Medications for chronic pain  Information about opioids  Information about cannabinoids |
| SUPPLEMENTAL: Sleep | Creating sleep logs  Initial steps to transform your sleep: Sleep stimulus control  Patient example  Sleep hygiene and behavioural recommendations that will improve your sleep |
| Final /Wrap up | Dealing with Setbacks  Recap of modules  “Rolling Down the River” mindfulness exercise |
